# Effects of circadian misalignment on cognition in chronic shift workers

**DOI:** 10.1038/s41598-018-36762-w

**Published:** 2019-01-24

**Authors:** Sarah L. Chellappa, Christopher J. Morris, Frank A. J. L. Scheer

**Affiliations:** 1 Medical Chronobiology Program, Division of Sleep and Circadian Disorders, Departments of Medicine and Neurology, Brigham and Women’s Hospital, Boston, MA 02115 USA; 2 Division of Sleep Medicine, Department of Medicine, Harvard Medical School, Boston, MA 02115 USA

## Abstract

Shift work is associated with increased human operational errors, presumably due to the circadian timing system that inhibits optimal cognitive function during the night. Circadian misalignment, which is the misalignment between the circadian pacemaker and behavioral/environmental cycles, impairs cognitive performance in non-shift workers. However, it remains uncertain whether the adverse cognitive consequences of circadian misalignment are also observed in chronic shift workers. Thus, we investigated the effects of circadian misalignment on cognitive performance in chronic shift workers. Using a randomized, cross-over design that simulated day shift work (circadian alignment) and night shift work (circadian misalignment), we show that circadian misalignment increases cognitive vulnerability on sustained attention, information processing and visual-motor performance, particularly after more than 10 hours of scheduled wakefulness. Furthermore, their increased levels of subjective sleepiness and their decreased sleep efficiency were significantly associated with impaired sustained attention and visual-motor performance. Our data suggest that circadian misalignment dramatically deteriorates cognitive performance in chronic shift workers under circadian misalignment. This increased cognitive vulnerability may have important safety consequences, given the increasing number of nighttime jobs that crucially rely on the availability of cognitive resources.

## Introduction

Shift work is typically referred to as an employment practice design to cover the 24-h day and night needed for duty performance in contemporary society^1,2^, and 14% of Americans perform their work during the night^3^. Night shift work is at odds with our endogenous circadian system, which is biologically tuned to ensure optimal cognitive performance during the biological day and promote sleep during the biological night^4^, even in chronic shift workers^5^. Indeed, evidence suggests that only a very small minority (<3%) of permanent night workers may have “complete” adjustment of their endogenous melatonin rhythm to night work (melatonin at low levels during the night shift, with the peak during daytime sleep), and that less than a quarter of permanent night workers evidence sufficient adjustment to derive any benefit from it^6–8^. Thus, permanent night-shift systems are unlikely to result in enough circadian adjustment in most individuals. This biological framework speaks against performing operational tasks at night and is a very likely explanation as to why key industry-related accidents during the last decades occurred during the middle of the night due to human errors^9,10^. Cognitive function, as indexed by working memory, visual-motor performance, information processing, sustained attention—to name a few—play a vital role in the performance of many operational tasks^11^. Thus, even a temporary failure of cognitive performance can jeopardize the ability to perform a given task, particularly when accurate and immediate responses are required^11^. Laboratory studies indicate that sleep loss—both acute and cumulative—decrease subjective perception of alertness and the ability to sustain attention^12,13^. Acute sleep restriction impairs a variety of cognitive tasks, particularly those that require the allocation of attentional resources, such as sustained, divided and selective attention^14–16^. Chronic partial restriction of sleep (i.e., 2–3 h sleep loss per night accumulated over multiple nights) can also result in deficits of performance (i.e., sustained attention), particularly when there is limited opportunity for sleep recovery^12,16–20^. Further reductions in sleep duration impair performance even more, e.g., on sustained attention^18^, and may be similar in magnitude to alcohol intake^21,22^. The relevance of these sleep-induced effects on cognitive function is that night shift workers may be chronically sleep restricted, partly because of circadian misalignment^5^. Circadian misalignment combined with extended wakefulness has been shown to decrease attention-based task performance during the first night shift^23^. More recently, we showed that repeated exposure to circadian misalignment (up to 3 consecutive days) adversely impacts a myriad of cognitive tasks, such as the psychomotor vigilance task performance (sustained attention), digit symbol substitution task (information processing), and unstable tracking task (visual-motor performance)^24^.

Surprisingly, very little is known about the consequences of circadian misalignment per se on cognition, as the overwhelming majority of studies focus on the effects of real-life shift work on operational errors and accidents^5,25,26^, on subjective measures of sleepiness/fatigue^27,28^ and on tasks related to attentional resources^29^. Importantly, real-life shift work studies cannot determine the independent effects of circadian misalignment on cognitive function in shift workers, due to e.g. differences in work performed during the day and night, environmental conditions between day and night shift work, among others. Thus, it remains uncertain whether the adverse cognitive consequences of circadian misalignment are also observed in chronic shift workers. Here, we investigated whether the adverse cognitive effects across different cognitive domains of circadian misalignment that has been shown in non-shift workers^24^ can be translated to chronic shift workers undergoing both a simulated night shift (circadian misalignment) and day shift (circadian alignment). Our main hypotheses are as follows:

1. Circadian misalignment impairs cognitive performance, as indexed by sustained attention, cognitive throughput, information processing and visual-motor performance, in chronic shift workers.
2. Circadian misalignment increases subjective levels of sleepiness in chronic shift workers, and their increased subjective sleepiness may be associated with increased performance impairment.
3. Circadian misalignment adversely impacts on sleep efficiency during the daytime sleep in chronic shift workers, and their decreased sleep efficiency is associated with increased performance impairment.

**Figure 1:**
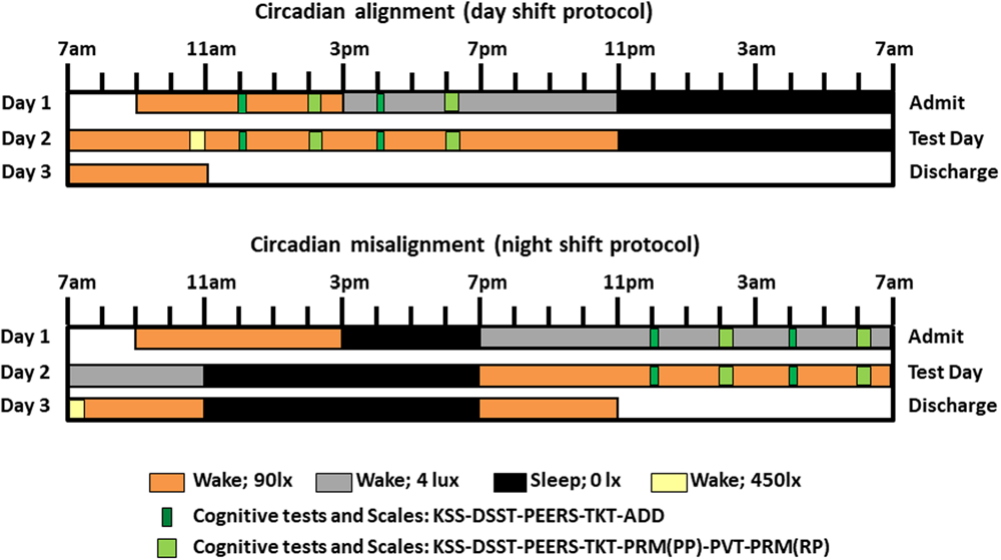
Within-subject, randomized, cross-over study design. Circadian alignment (upper panel) and misalignment (lower panel) protocols. For the former, scheduled sleep times were maintained between 11PM to 7AM across all days, while for the latter these timings were inverted by 12 h after Day 1. During Day 2 for the aligned condition, the Psychomotor Vigilance Task (PVT) and Probed Recall Memory (PRM) with Presentation phase (PP) and Recall phase (RP) were conducted at 2PM and 6PM, the Addition Task (ADD) at 12PM and 4PM, and the Unstable Tracking Task (TKT), Digit Symbol Substitution Task (DSST), Performance evaluation and effort scales (PEERS) and Karolinska Sleepiness Scale (KSS) at 12PM, 2PM, 4PM and 6PM. For the misaligned protocol, timing of cognitive testing during Day 2 was inverted by 12 h. Light levels were 90 lux to simulate typical room light intensity, 450 lux for 30-minute periods to simulate the morning commute preceding the simulated day shift and following the simulated night shift, 4 lux to permit assessment of dim-light melatonin levels, and 0 lux during scheduled sleep.

To test these hypotheses, cognitive tests encompassing sustained attention, cognitive throughput, information processing, visual-motor performance and declarative memory, as well as subjective scales of sleepiness and performance ratings, were conducted during scheduled waketime (Fig. 1).

## Results

**Figure 2:**
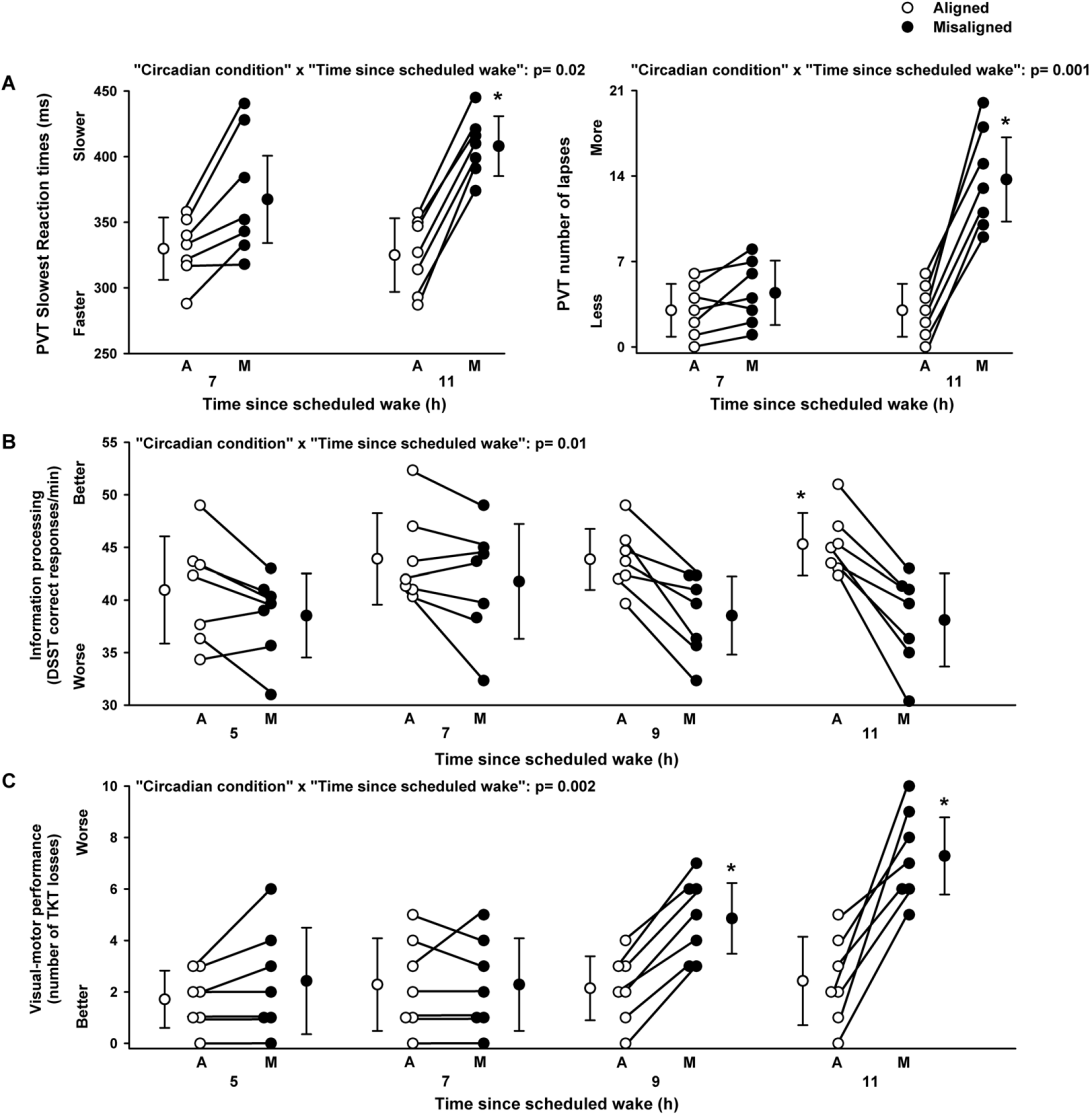
Cognitive performance in chronic shift workers under circadian alignment/misalignment. (**A**) Sustained attention (PVT 10% slowest reaction times and PVT lapses) worsened under circadian misalignment, particularly after 11 h of scheduled wakefulness, as compared to circadian alignment. (**B**) Information processing (number of correct DSST responses/min) performance improved *only* under circadian alignment, particularly when DSST assessments occurred 11 h after scheduled wakefulness, as compared to circadian misalignment. (**C**) Visual-motor performance (number of TKT losses) became progressively worse under circadian misalignment, particularly after 7 h of scheduled wakefulness, as compared to circadian alignment. White and black circles correspond to individual data under circadian alignment and misalignment conditions, respectively. Data correspond to mean ± standard error of the mean, *p < 0.05 (see results for statistics).

PVT performance (slowest reaction times and number of lapses) significantly varied by the main effect “circadian alignment/misalignment condition” (F_1,18_ = 3.5, *p* = 0.04; F_1,18_ = 7.3, *p* = 0.02, respectively), and by the interaction of “circadian alignment/misalignment condition” and “time since scheduled wake” (F_1,18_ = 6.6, *p* = 0.02; F_1,18_ = 15.4, *p* = 0.001, respectively). A cognitive slowing occurred when individuals were under circadian misalignment, as compared to circadian alignment, particularly 11 h since scheduled waketime (multiple post-hoc Bonferroni correction, *p* < 0.05) (Fig. 2A). Performance to the mathematical addition task, as indexed by the participant’s ability to accurately sum two-digit numbers (number of correct responses per minute), did not significantly vary by condition (F_1,18_ = 0.27, *p* = 0.61) nor by the interaction of “circadian alignment/misalignment condition” and “time since scheduled wake” (F_1,18_ = 0.01, *p* = 0.93) (data not shown). Information processing (DSST correct responses per minute) did not significantly vary by the main effect “circadian alignment/misalignment condition” (F_1,46_ = 0.1, *p* = 0.33). Conversely, we observed a significant interaction of “circadian alignment/misalignment condition” and “time since scheduled wake” (F_1,46_ = 6.6, *p* = 0.01), such that only under circadian alignment DSST performance improved over time since scheduled wake, particularly 11 h since scheduled waketime (multiple post-hoc Bonferroni correction, *p* < 0.05) (Fig. 2B). Visual-motor performance (number of Unstable Tracking Task losses) significantly varied by the main effect “circadian alignment/misalignment condition” (F_1,40_ = 4.4, *p* = 0.04), and by the interaction of “circadian alignment/misalignment condition” and “time since scheduled wake” (F_1,46_ = 10.6, *p* = 0.002). Accordingly, the number of losses remained stable when individuals were under circadian alignment, while it progressively increased (worsened) when the same individuals were misaligned beyond 7 h of scheduled wakefulness (multiple post-hoc Bonferroni correction, *p* < 0.05) (Fig. 2C). Declarative memory (Probed recall memory Task, % correct responses) did not vary by the main effect “circadian alignment/misalignment condition” (F_1,18_ = 0.1, *p* = 0.34), nor by the interaction of “circadian alignment/misalignment condition” and “time since scheduled wake” (F_1,46_ = 0.2, *p* = 0.39), with similar performance levels when individuals were under either circadian conditions (data not shown).

**Figure 3:**
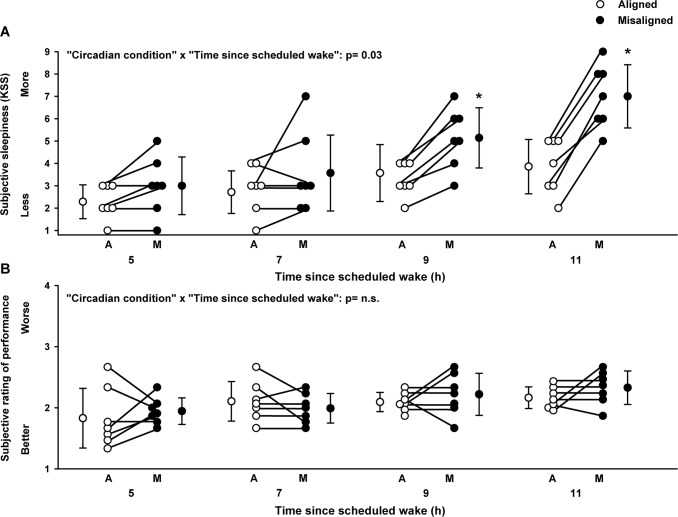
Subjective ratings of sleepiness and performance in chronic shift workers under circadian alignment/misalignment. (**A**) Subjective sleepiness (KSS) indicated higher levels of sleepiness during circadian misalignment, particularly after 9 h of scheduled wakefulness, as compared to circadian alignment. (**B**) Subjective ratings of performance (PEERS) did not differ between circadian alignment and misalignment conditions. White and black circles correspond to individual data under circadian alignment and misalignment conditions, respectively. Data correspond to mean ± standard error of the mean, *p < 0.05 (see results for statistics).

**Figure 4:**
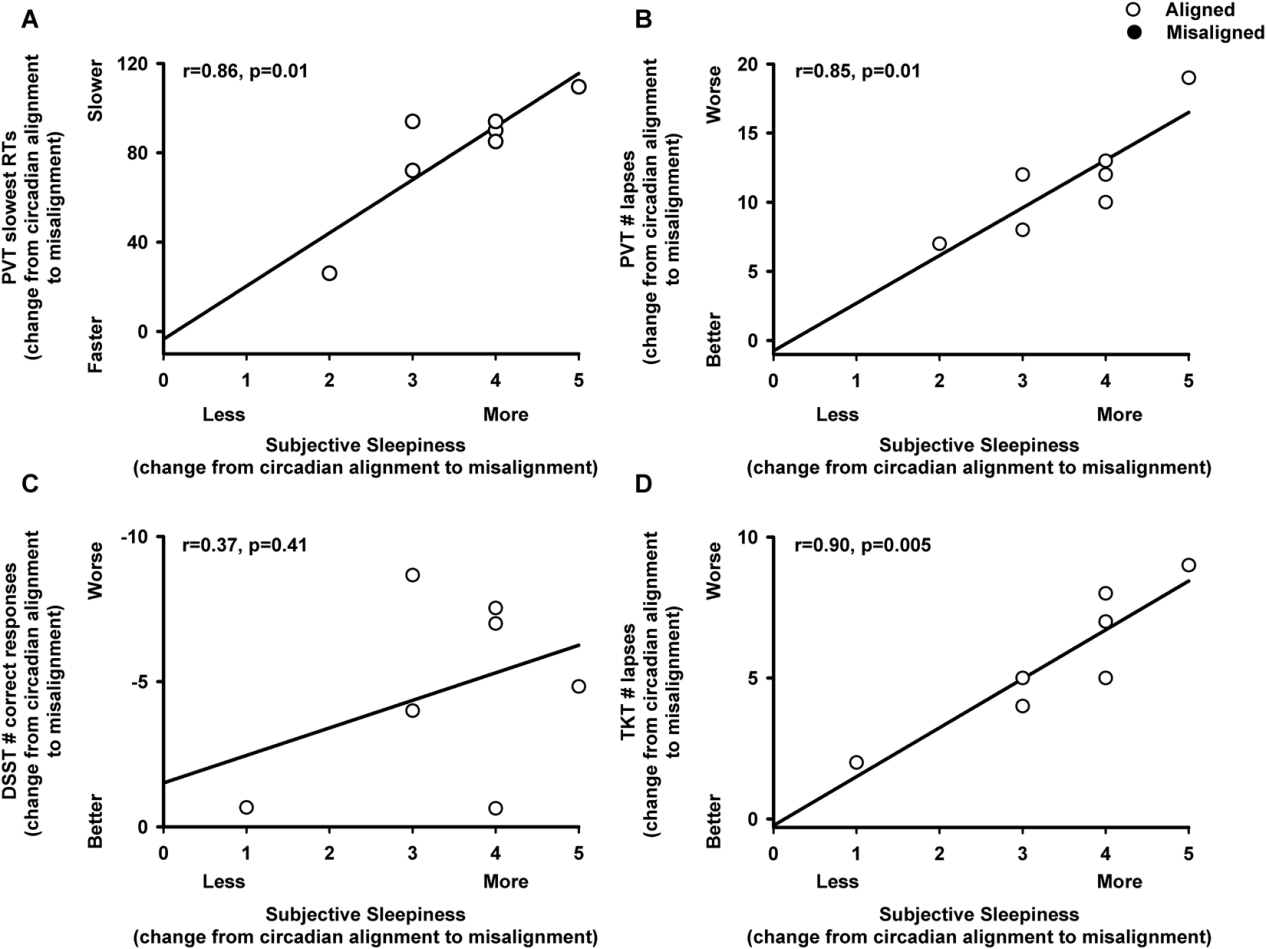
Association of misalignment-induced change in subjective sleepiness with cognitive performance in chronic shift workers. (**A**,**B**) Correlations between effect of misalignment for subjective sleepiness levels (x-axis: KSS levels) and for sustained attention (y-axis: (**A**) PVT slowest reaction times, (**B**) PVT lapses), (**C**) information processing (y-axis: DSST ratio of number of correct responses/minute), and (**D**) visual-motor performance (y-axis: TKT number of losses). X-axis and Y-axis correspond to the change from circadian alignment to misalignment conditions (see results for statistics).

Subjective levels of sleepiness significantly varied by the main effect “circadian alignment/misalignment condition” (F_1,46_ = 4.6, *p* = 0.04), and by the interaction of “circadian alignment/misalignment condition” and “time since scheduled wake” (F_1,46_ = 4.9, *p* = 0.03). Accordingly, subjective sleepiness was higher when individuals were under circadian misalignment, as compared to they were under circadian alignment, beyond 7 h since scheduled waketime (multiple post-hoc Bonferroni correction, *p* < 0.05) (Fig. 3A). Conversely, subjective ratings of performance did not significantly vary by the main effect “circadian alignment/misalignment condition” (F_1,46_ = 0.1, *p* = 0.76), nor by the interaction of “circadian alignment/misalignment condition” and “time since scheduled wake” (F_1,46_ = 0.35, *p* = 0.56), with similar subjective ratings of performance between both circadian conditions (Fig. 3B). Importantly, subjective ratings of performance were not significantly associated with any cognitive measures (*p* > 0.1). In contrast, more subjective sleepiness (change from circadian alignment to misalignment conditions) was significantly associated with deficits in sustained attention (change from circadian alignment to misalignment conditions, Pearson product moment correlation, PVT slow reaction times: *r* = 0.86, *p* = 0.01; PVT lapses: *r* = 0.85, *p* = 0.01) and visual-motor performance processing (change from circadian alignment to misalignment conditions, Pearson product moment correlation, TKT number of losses: *r* = 0.90, *p* = 0.004) (Fig. 4).

**Figure 5:**
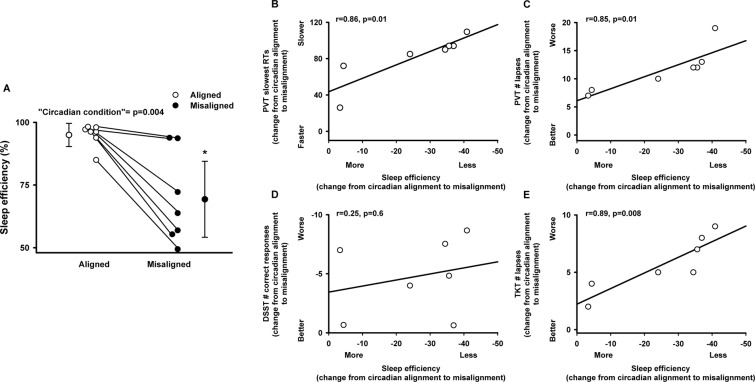
Association of sleep efficiency with cognitive performance in chronic shift workers. (**A**) Sleep efficiency was lower during circadian misalignment, as compared to when the same individuals were under circadian alignment. Correlations between sleep efficiency levels (x-axis: difference of sleep efficiency between circadian misalignment and alignment conditions) and sustained attention (y-axis: (**B**) PVT slowest reaction times, (**C**) PVT lapses), (**D**) information processing (y-axis: DSST ratio of number of correct responses/minute), and (**E**) visual-motor performance (y-axis: TKT number of losses). X-axis and Y-axis correspond to the difference between circadian misalignment and alignment conditions. For panel A, white and black circles correspond to individual data under circadian alignment and misalignment conditions, respectively. Data correspond to mean ± standard error of the mean, *p < 0.05 (see results for statistics).

Lastly, we investigated whether prior sleep, as indexed by polysomnographically determined sleep efficiency in the sleep episode preceding the scheduled wake episode with cognitive tests, was associated with performance. Sleep efficiency significantly varied by the main effect “circadian alignment/misalignment condition” (F_1,6_ = 21.6, *p* = 0.004), as previously shown^30^, such that lower sleep efficiency was observed when individuals were under circadian misalignment (daytime sleep). Furthermore, misalignment-induced decreased sleep efficiency (change from circadian alignment to misalignment conditions) correlated significantly with impaired sustained attention (change from circadian alignment to misalignment conditions, Pearson product moment correlation, PVT slow reaction times: *r* = 0.86, *p* = 0.01; PVT lapses: *r* = 0.85, *p* = 0.01), and with visual-motor performance (change from circadian alignment to misalignment conditions, Pearson product moment correlation, TKT number of losses: *r* = 0.89, *p* = 0.008) (Fig. 5).

## Discussion

Our data indicate deteriorated performance in tasks associated with sustained attention, information processing and visual-motor performance in chronic night shift workers under circadian misalignment, particularly after 11 hours of scheduled wakefulness. Furthermore, their subjective perception of sleepiness and their prior sleep efficiency were associated with sustained attention and visual-motor performance. These data suggest that when chronic shift workers are under circadian misalignment their performance may dramatically deteriorate if exposed to longer durations of wakefulness.

In a similar vein as for our previous controlled laboratory study, which also used a within-subject, randomized crossover design including circadian misalignment and alignment protocols in non-shift workers^24^, the circadian misalignment effects on cognitive performance showed a task-dependency in chronic shift workers. Here, we observed that chronic shift workers exposed to circadian misalignment showed deficits in sustained attention (PVT), information processing speed (DSST), and increased lapses in the visual-motor task (TKT). By contrast, accuracy in a simple mathematical addition task (ADD) and declarative memory performance (PRM) were virtually identical between circadian conditions. Thus, our results in chronic shift workers are consistent with the acute effects of circadian misalignment (first day after the “slam shift”) in non-shift workers^24^, suggesting that even in chronic shift workers, circadian misalignment negatively impacts cognitive performance. Some hypotheses have been proposed to explain why specific aspects of cognitive performance are vulnerable to circadian misalignment and/or sleep loss, most of which associated with general effects on alertness and attention^31–33^. Cognitive impairments would be mediated through decreased alertness and attention through lapses, slowed responses, and wake-state instability^33^. These deficits in performance would be most likely to deteriorate in long, simple, and monotonous tasks requiring reaction, speed or vigilance, plausibly due to fluctuations in alertness^33^. Importantly, two aspects of performance are of critical importance: speed and accuracy. In principle, individuals can switch their emphasis between the two with attentional focusing^34^. Oftentimes, concentrating on improving one aspect leads to the deterioration of the other. This is called the speed/accuracy trade-off phenomenon^31,34^. Laboratory studies involving sleep deprivation and/or circadian misalignment have found cognitive impairments mostly on performance speed (i.e., visual search tasks, DSST and/or TKT), whereas accuracy (i.e., working memory and/or declarative memory performance) may remain intact, particularly for self-paced tasks^24,35,36^. A neurobiological explanation for this decreased speed and/or increased errors when individuals are misaligned or have accrued sleep debt may be due to changes in local neuronal activity (as indexed by local field potentials, LFP), which become attenuated, delayed, and lengthened just before cognitive (PVT) lapses^37^. Furthermore, LFPs showed increased delta/theta EEG activity, which in turn was associated with degraded neuronal responses and decreased cognitive performance^37^. These data suggest that cognitive lapses may involve local fluctuations in hallmarks of alertness (i.e., EEG) and in underlying neuronal activity (i.e., LFP). Collectively, these laboratory studies may provide a rationale as to why tasks tapping onto the allocation of attentional resources and speed may be more jeopardized when individuals perform at night, as compared to during the day.

Our data indicate that maximal cognitive vulnerability occurred in a time window surrounding 9–11 h of scheduled wakefulness (4–6AM). Data from stringently controlled circadian protocols (e.g., forced desynchrony and constant routine) indicate decreased sustained attention, information processing, visual-motor performance, working memory and higher-order cognitive function across wakefulness, particularly when individuals perform during the biological night (interaction between time since wake and circadian phase)^38–41^. Underlying mechanisms include a strong circadian drive for sleep in the late biological night/early morning hours (e.g., ~3:00–6:00 AM in diurnal humans)^4^ and increased homeostatic sleep pressure – if sleep is curtailed or partially disrupted^20^. This day-night circadian variation in sleep-wake propensity is a likely candidate for the jeopardized alertness and performance levels of workers who try to remain awake at night to work.

Night shift workers show impaired working memory, processing speed and cognitive flexibility *after* a night shift^42^, and more operational errors, occupational accidents and injuries, as compared to day shift workers^5,43^. We observed that, in chronic shift workers who are misaligned, increased sleepiness levels and reduced sleep efficiency (sleep prior to their cognitive testing) were associated with impaired sustained attention and visual-motor performance, which heavily depend on an individual’s vigilance state^39,44^. Epidemiological data suggest that night shift workers show increased levels of subjective sleepiness and decreased vigilant attention performance when they work consecutive 12-h shifts^45^. Collectively, the real-life implication for these findings is that subjective sleepiness levels in night shift workers may be related to some aspects of their cognitive performance – such as reaction times, lapses and errors. Operator distraction is a major safety concern, as it compromises safety by taking the operator’s attention away from their operational environment^5^. Laboratory research show that distractibility and errors markedly increase when individuals are sleepy, particularly at night^46,47^, thus pointing to an association between subjective sleepiness and objective performance.

Limitations in our study include few cognitive test sessions during wakefulness, due to other measurements (i.e., metabolic and cardiovascular outcomes) that were also conducted during the study^30,48^. Furthermore, the low study sample may limit generalizability to shift workers. Thus, future studies with larger sample sizes are needed to confirm the generalizability of our findings to real-world settings.

In summary, our data indicate that in chronic shift workers circadian misalignment causes deterioration of performance in tasks associated with sustained attention, information processing and visual-motor performance, particularly when they were exposed to longer durations of wakefulness. We also observed that the increased subjective sleepiness and decreased sleep efficiency experienced by chronic shift workers under circadian misalignment associated with impaired cognitive hallmarks of attentional resources, which may pose a risk for human operational errors. Importantly, we show that chronic shift workers may not necessarily adapt to shift work due to years of shift work experience, or be selected to be resilient against the adverse effects of circadian misalignment on cognitive function. The increased cognitive vulnerability described in our study may have important safety consequences, given the increasing number of jobs performed at night that are highly dependent on the availability of cognitive resources.

## Methods

Other results from this protocol, addressing independent hypotheses, have been published^30,48^.

### Participants

Research participants gave written informed consent, and the study was approved by the Partners Human Research Committee and performed in accordance to the Declaration of Helsinki. Nine, healthy, non-smoking, drug- and medication-free (except for oral contraceptives) adults completed this study (for more details, see^30,48^). Two participants’ dim light melatonin onset (DLMOn) showed a phase difference >4 h between the circadian alignment and misalignment protocols^30,48^. Data from these two participants were excluded from all subsequent analysis as they had an unstable timing of their central circadian clock as done before^30,48^. For the remaining 7 participants, their demographics were as follows: age 37 ± 7 years old [30–48 years old], sex 3 men and 4 women, BMI 24.4 ± 3.1 kg/m^2^ [21.0–29.3 kg/m^2^], night work frequency 12 ± 4 night shifts/month [6–18 night shifts/month], consecutive shift work experience, 5.3 ± 8.8 years [1.3–25.1 years], lifetime cumulative shift work experience 6.3 ± 8.5 years [1.5–25.1 years].

### Study design

For ≥14 days before each laboratory visit, participants wore an Actiwatch Spectrum (Philips-Respironics, Murrysville PA), recorded their bedtimes, wake times and work schedules in a diary, and reported the information to a time-stamped voicemail system. Participants were instructed to sleep between 11PM and 7AM on the night preceding each inpatient admission to reduce possible sleep debt before entering the laboratory.

Each participant underwent a within-subject, randomized crossover study that comprised two 3-day laboratory protocols (Fig. 1). One protocol included a simulated day shift (circadian alignment) protocol and the other a simulated night shift (circadian misalignment) protocol. The visits were separated by 3–8 weeks (mean ± SD: 5 ± 2 weeks). Minimization was used to reduce imbalance, according to age, sex and BMI. Participants remained in a private laboratory room throughout each laboratory protocol to allow strict control of environmental conditions. Participants were not permitted to exercise while in the laboratory. In the circadian alignment protocol, participants’ sleep opportunity occurred from 11PM until 7AM for days 1–3. In the circadian misalignment protocol, on day 1 the participants’ sleep/wake cycle was inverted by 12 h by including an 8-h wake episode (beginning at the scheduled time of awakening of 7AM on day 1 at home) plus a 4-h sleep opportunity between 3PM and 7PM, to maintain a 2:1 ratio between scheduled wakefulness and sleep opportunity. The subjects then stayed awake for 16 h until their next 8-h sleep opportunity that occurred from 11AM until 7PM. This sleep/wake cycle was maintained until the end of the protocol (day 3). Light levels during both protocols are shown in Fig. 1.

### Cognitive performance and subjective scales

Cognitive tests occurred 5–11 h since scheduled waketime to avoid sleep inertia effects on cognitive performance^38^. Cognitive domains of interest included sustained attention, cognitive throughput, information processing, visual-motor function and declarative memory, due to their sensitivity to the cumulative effects of adverse circadian phase and increased sleep pressure^44,49^. Only data from the test days were used in the final analysis. Data from the first full wake episode of tests were included to minimize a first exposure or learning effect on the results.

The Psychomotor Vigilance Task (PVT) is a sustained attention task sensitive to sleep loss and circadian rhythmicity^12,13^ and we assessed the slowest PVT reaction times and PVT lapses^18,50^. The visual serial addition task (ADD) corresponds to a mathematical addition task that involves working memory, attention and arithmetic capabilities^40^. Here, we used the correct sum of pairs as an index for accuracy to task. The Digit Symbol Substitution Task (DSST) is a test of information processing^41,51^, and the ratio of correct responses/minute was used to index their performance. The unstable tracking task (TKT) is a visual-motor performance task, which indexes operational error^24,52^, and we assessed the number of losses to index their performance. The probed recall memory (PRM) is a test of declarative memory for unassociated pairs of words that varies with both scheduled waketime and circadian phase^40^, and we assessed the percentage of correct responses during recall (10-min delay). For detailed information on each cognitive task, see^24^. Subjective sleepiness was assessed with the Karolinska Sleepiness Scale (KSS)^53^ and subjective rating of performance was indexed by the likert-based Performance Evaluation and Effort Rating Scales (PEERS)^24,54^. The order of presentation for the cognitive test battery and subjective scales was fixed across all participants under both circadian conditions and was as follows: at 5 h and 9 h after scheduled waketime, task presentation was KSS-DSST-PEERS-TKT-ADD; and at 7 h and 11 h after scheduled waketime task presentation was KSS-DSST-PEERS-TKT-PRM (presentation phase)-PVT-PRM (recall phase).

### Polysomnography

Sleep was recorded by polysomnography (Vitaport; TEMEC Instruments), in accordance with the American Academy of Sleep Medicine recommendations^55^, during the first sleep opportunity in the circadian alignment protocol and during second sleep opportunity in the circadian misalignment protocol. Sleep stages were scored visually per 30-s epochs, according to^55^, by a single experienced polysomnography technician, blind to the circadian alignment/misalignment conditions.

### Data analysis and statistics

All statistical analyses were performed with SAS version 9.3 (SAS Institute, Cary, NC, USA). Cognitive data (PVT, DSST, ADD, TKT and PRM) and subjective data (KSS and PEERS) for Day 2 in the study protocols were used in the analyses (to avoid practice effects during Day 1). All data were normalized using the TRANSREG approach (PROC TRANSREG, SAS)^24^. Accordingly, the cognitive data were normalized as follows: PVT analyses on log-transformed slowest reaction times and lapses, ADD analyses on the number of correct responses, DSST analyses on the ratio of number of correct responses per minute, TKT analyses on the log-transformed number of losses, PRM analyses on log-transformed number of correctly recalled word pairs. For the subjective scales, KSS analyses were performed on log-transformed subjective sleepiness data, and PEERS analyses on the raw data of performance ratings. To examine the time-course of cognitive performance and subjective scales, comparisons were made with linear mixed-model analyses of variance for repeated measures (PROC MIXED, SAS), with main factors “circadian alignment/misalignment condition” and “time since scheduled wake” (PVT and PRM: 7 h and 11 h, ADD: 5 h and 9 h, and TKT, DSST, KSS and PEERS: 5 h, 7 h, 9 h and 11 h), and their two-way interaction. We then computed post-hoc multiple comparisons test for this specific interaction, which were adjusted for multiple comparisons using Tukey-Kramer corrections on α ≤ 0.05. “Participant” was included as a random factor. Contrasts were assessed with the LSMEANS statement. We also performed correlations (Pearson product moment correlation) to identify whether subject ratings of sleepiness and performance are associated with cognitive performance under circadian alignment and misalignment conditions. Furthermore, we tested whether the ability to sleep (sleep efficiency) is associated with cognitive performance under both circadian conditions by using correlations (Pearson product moment correlation). Data are presented as mean ± SEM.

## Data Availability

The datasets generated during the current study are available from the corresponding authors upon reasonable request.
